# The role of multimodal imaging in characterization and monitoring of choroidal neovascularization secondary to angioid streaks

**DOI:** 10.1177/11206721241257976

**Published:** 2024-05-27

**Authors:** Adnan Kilani, Denise Vogt, Armin Wolf, Efstathios Vounotrypidis

**Affiliations:** 1 Department of Ophthalmology, Ulm University, Prittwitzstraße 43, 89075 Ulm, Germany

**Keywords:** Angioid streaks, pseudoxanthoma elasticum, choroidal neovascularization, spectral-domain optical coherence tomography angiography, spectral-domain optical coherence tomography, fluorescein angiography

## Abstract

**Background:**

To characterize and monitor choroidal neovascularisation (CNV) secondary to angioid streaks (AS) using multimodal imaging and to compare the results with conventional fluorescein angiography (FA).

**Methods:**

A total of 11 eyes with CNV secondary to AS were included in this retrospective study. Multimodal morphological and functional assessment, including spectral-domain optical coherence tomography (SD-OCT), spectral-domain optical coherence tomography angiography (SD-OCTA), and fundus autofluorescence (FAF), were used to assess for evidence of CNV activity and compared with conventional FA. Morphological features of CNV were analyzed and treatment was continuously monitored using SD-OCT and SD-OCTA.

**Results:**

Our results showed that SD-OCTA provided reliable results for the detection of secondary CNV in AS that were comparable to conventional FA. With SD-OCTA, a total of 13 CNVs were detected in 11 eyes and analyzed by means of outer retinal choriocapillaris depth (ORCC) segmentation and the corresponding B-scans. Twelve of the 13 CNVs were classified as active and therefore required treatment. For treatment monitoring during intravitreal therapy (IVT), SD-OCTA was found to be a valuable diagnostic tool over a mean follow-up of 76 weeks.

**Conclusions:**

Our study demonstrates that SD-OCTA can be routinely used to identify ill-defined CNV without dye-based angiography, especially in cases of CNV secondary to AS, where Bruch's membrane (BM) defects limit the diagnostic value of FA. Our results showed that non-invasive multimodal imaging facilitates sufficient CNV monitoring and treatment guidance. Further studies are warranted to provide more evidence in this rare retinal disease.

## Introduction

Angioid streaks (AS) are bilateral breaks in the Bruch's membrane (BM) and appear clinically as vessel-like structures radiating from the optic disc.^1–3^ Histopathological findings include calcium deposits in the BM layer with corresponding atrophy of the retinal pigment epithelium (RPE).^1,3–5^ More than 50% of patients with AS suffer from systemic diseases, including pseudoxanthoma elasticum (PXE), Paget's disease, beta-thalassemia, or sickle cell disease, with PXE being the most common.^1,3,6–9^

Ophthalmologically, patients with AS are often initially asymptomatic, but have a high risk (approximately 70–80%) of developing secondary choroidal neovascularization (CNV).^1,10–12^ Increased BM calcification causes a permeability disorder between RPE and choroid, leading to an accumulation of degradation products that induce hypoxia in the RPE and constantly promote the development of CNV.^13^ Based on the pathophysiology, CNV secondary to AS are prone to be refractory to therapy and recurrent.^3,10,11,13,14^ Therefore, reliable detection of CNV is essential for potentially appropriate therapy with anti-VEGF.^3,10,11^

The diagnosis of AS is primarily clinical using fundoscopy. However, concomitant findings such as CNV can be better visualized with multimodal imaging, including fluorescein angiography (FA), spectral-domain optical coherence tomography angiography (SD-OCTA), infrared and red-free fundus imaging, fundus autofluorescence (FAF) and spectral-domain optical coherence tomography (SD-OCT).^1–3,10,15–20^ In AS, SD-OCT imaging is particularly important to detect any secondary CNV, including activity biomarkers such as subretinal hyper-reflective material (SHRM) as a biomarker of CNV activity.^12,17,19,21–24^

Therefore, the aim of this study was to evaluate multimodal imaging features of CNV secondary to AS and to compare the results with conventional FA. Continuous treatment monitoring was analyzed during follow-up using SD-OCT and SD-OCTA.

## Material and methods

In this retrospective longitudinal case series, a total of eleven eyes of six patients with CNV secondary to AS were evaluated and treated at the Department of Ophthalmology, University Hospital of Ulm, Germany, between February 2016 and October 2020. Inclusion criteria were the presence of CNV secondary to AS detected by FA, SD-OCTA, and presence of typical SD-OCT findings (sub/intraretinal fluid, sub-RPE fluid or retinal pigment epithelium detachment [PED]). On SD-OCTA, the presence of CNV was defined as the evidence of a vascular decorrelation signal located directly above or below the RPE (type 2 or 1 CNV, respectively). For CNV detection by OCTA, SD-OCT angiograms (3 × 3 mm and 6 × 6 mm) were obtained by outer retinal chorio-capillaris (ORCC) segmentation and corresponding B-scans with positive blood flow registration. The ORCC segmentation spans the outer plexiform layer, the outer nuclear layer and the choriocapillaris (20 µm below the RPE) and is preferably applied as a screening slab for type 1 or 2 CNV.^25^ CNV classification according to FA, classic CNV was defined as a bright, lacy, and well-defined hyperfluorescent lesion that appears in the early phase of FA and progressively leaks in the late phase. Occult CNV was categorized as fibrovascular PED or late leakage of undetermined source.^26,27^

Exclusion criteria were optic media opacities, ammetropia more than −6 diopters or +3 diopters or axial length more than 27 mm or less than 22 mm, other retinal-choroidal diseases, intraocular surgery in the previous four months and the absence of secondary CNV due to other retinal pathologies.

The study was conducted in accordance with the tenets of the Declaration of Helsinki and was approved by the local ethics committee (application number 388/15). Written informed consent was obtained from each subject prior to enrolment.

### Baseline examinations

All participants underwent a standard ophthalmic examination, including a medical history assessment, slit-lamp examination and fundoscopy after pupil dilation. Multimodal imaging consisted of the following modalities: Color fundus photography (CFP), FAF, FA (Zeiss FF450PlusIR fundus camera, CLARUS 500, Carl Zeiss Meditec Inc.), SD-OCT (Heidelberg Spectralis OCT, Heidelberg Engineering Inc. or Cirrus 5000 HD-OCT, Carl Zeiss Meditec Inc.) and SD-OCTA (3 × 3 mm and 6 × 6 mm; Zeiss Cirrus 5000 HD-OCT with AngioPlex™ module, Carl Zeiss Meditec Inc., software version 9.5.1.13585).

Multimodal imaging findings were independently evaluated by two experienced readers (A.K., E.V.). In case of uncertainty a third one (D.V.) was consulted. In addition to the above method of classification by anatomical location, CNV activity was classified as active or inactive using SD-OCT. This was complemented by comparative analysis of corresponding B-scans from SD-OCTA. An active lesion was suspected if any of the following criteria were present: (1) subretinal hyperreflective material (SHRM), (2) intra- and/or (3) subretinal fluid compartments.

Subsequently, all patients underwent cardiovascular and hematological evaluation. Furthermore, a human genetic work-up was recommended for all patients, which was also performed with human genetic counseling upon consent. Pseudoxanthoma elasticum (PXE) was strongly suspected in the presence of the following ophthalmological pathognomonic signs AS, peau d’orange (confluent yellowish RPE lesions, usually located temporal area of the macula) and the so-called “comet lesions’’ (small, round chorioretinal atrophy in the midperiphery of the retina, resembling comet-like lesions).^9,28^ The diagnosis of PXE was confirmed using the Plomp criteria.^9^ Therefore, in the presence of pathognomonic ophthalmological findings such as peau d'orange and AS, despite the absence of dermatological signs of PXE disease, a human genetic (to detect a homozygous pathogenic deletion in the ABCC6 gene) as well as cardiovascular work-up was mandatory.^9^

### Follow-up examinations and therapy

Intravitreal treatment (with either bevacizumab or ranibizumab) was performed at baseline and patients were retreated with a pro-re-nata (PRN) regimen, based on visual acuity and CNV activity on SD-OCT scans.

## Results

A total of eleven eyes of six patients with AS-related CNV were included in this study. A detailed description of all clinical and multimodal imaging features is provided, with particular emphasis on the characterization and management of secondary CNV using SD-OCT and SD-OCTA in correlation to conventional FA. Continuous multimodal treatment monitoring was analyzed over a mean follow-up of 76 weeks.

### Study population at baseline

AS were clinically diagnosed by fundoscopy in two female and four male subjects with a median age of 48 years (range min-max, 40 to 71 years). Nine eyes were phakic and one patient was pseudophakic in both eyes. The pseudophakic patient was previously treated with intravitreal anti-VEGF regimen in both eyes. The remaining eyes were untreated at baseline. Mean visual acuity at baseline was 0.49 ± 0.45(SD) logMAR.

**Figure 1.**
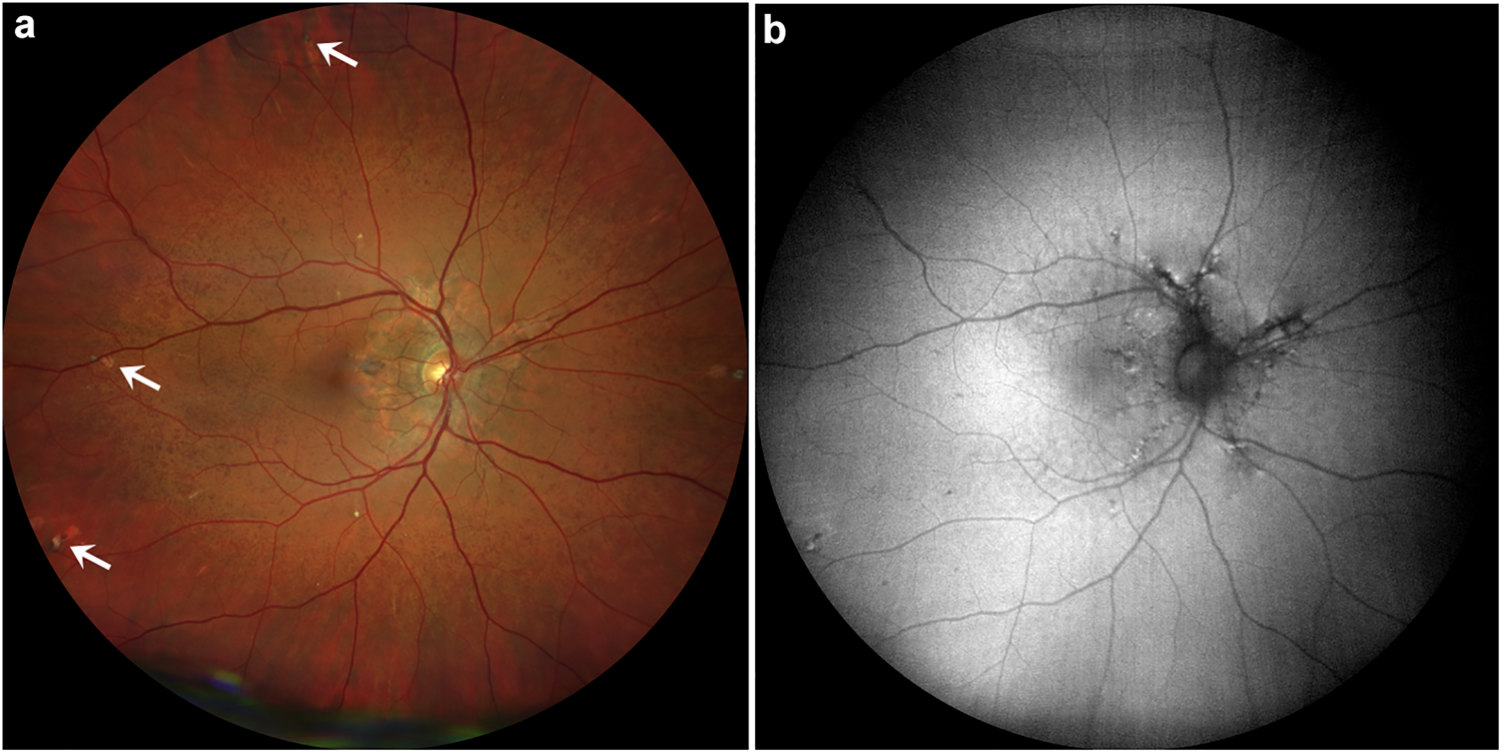
Right eye with AS, peau d'orange and comet lesions of study patient number 4 with confirmed pseudoxanthoma elasticum (PXE) disease. (a) Fundus with AS, peau d'orange and comet lesions (white arrows). (b) Corresponding fundus autofluorescence (FAF).

**Figure 2.**
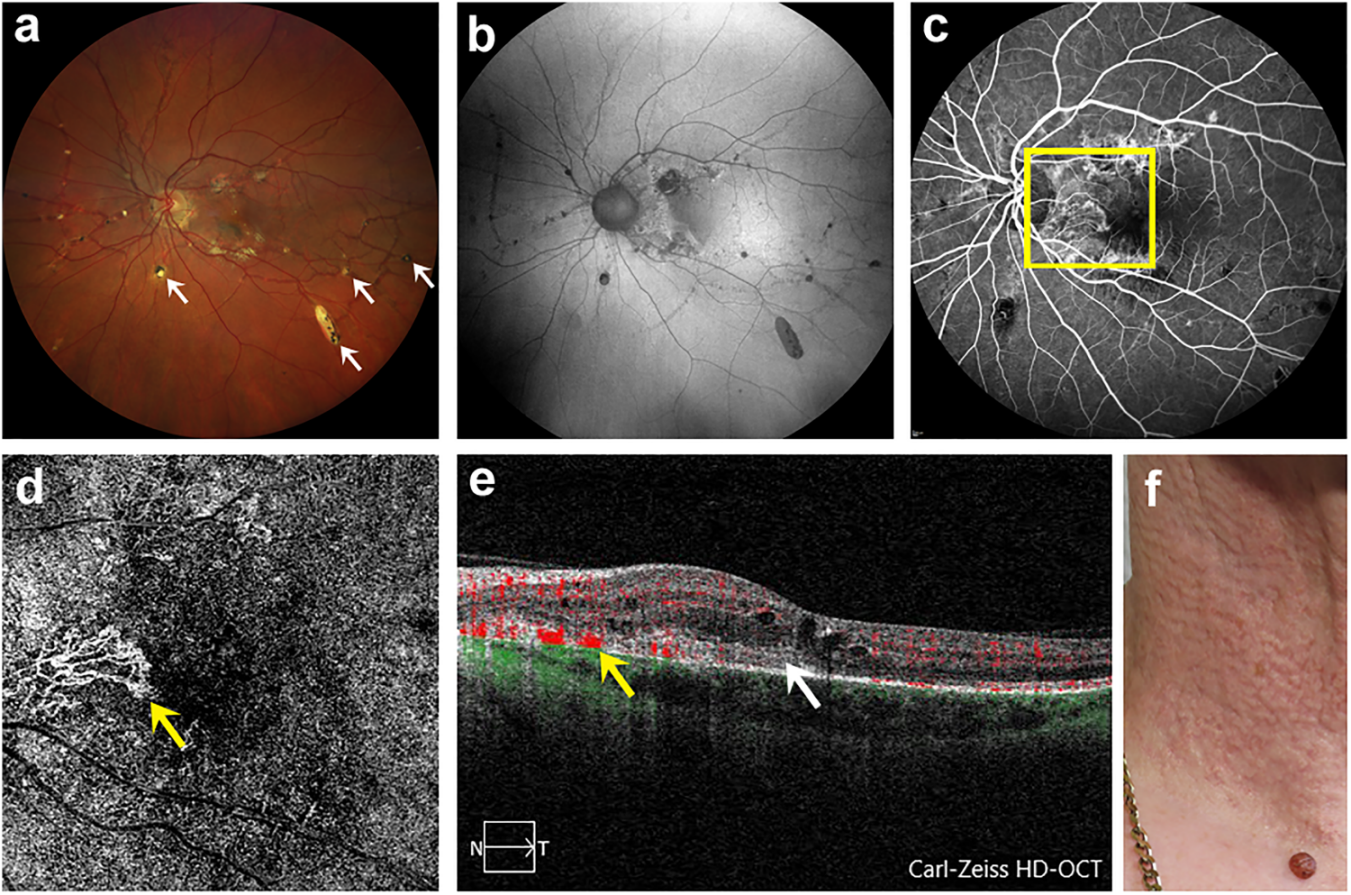
Left eye with AS, macular CNV and comet lesions of study patient number 6 with confirmed PXE disease and typical efflorescences. (a) Fundus with AS and comet lesions (white arrows). (b) Corresponding FAF. (c) FA showing macular CNV (yellow square). (d) SD-OCTA image in ORCC segmentation allowing a detailed visualization of macular CNV (yellow arrow). (e) Corresponding B-scan of macular CNV with blood flow registration (yellow arrow) and SHRM (white arrow). (f) Photograph of affected neck region showing flat, symmetrically arranged papules.

**Figure 3.**
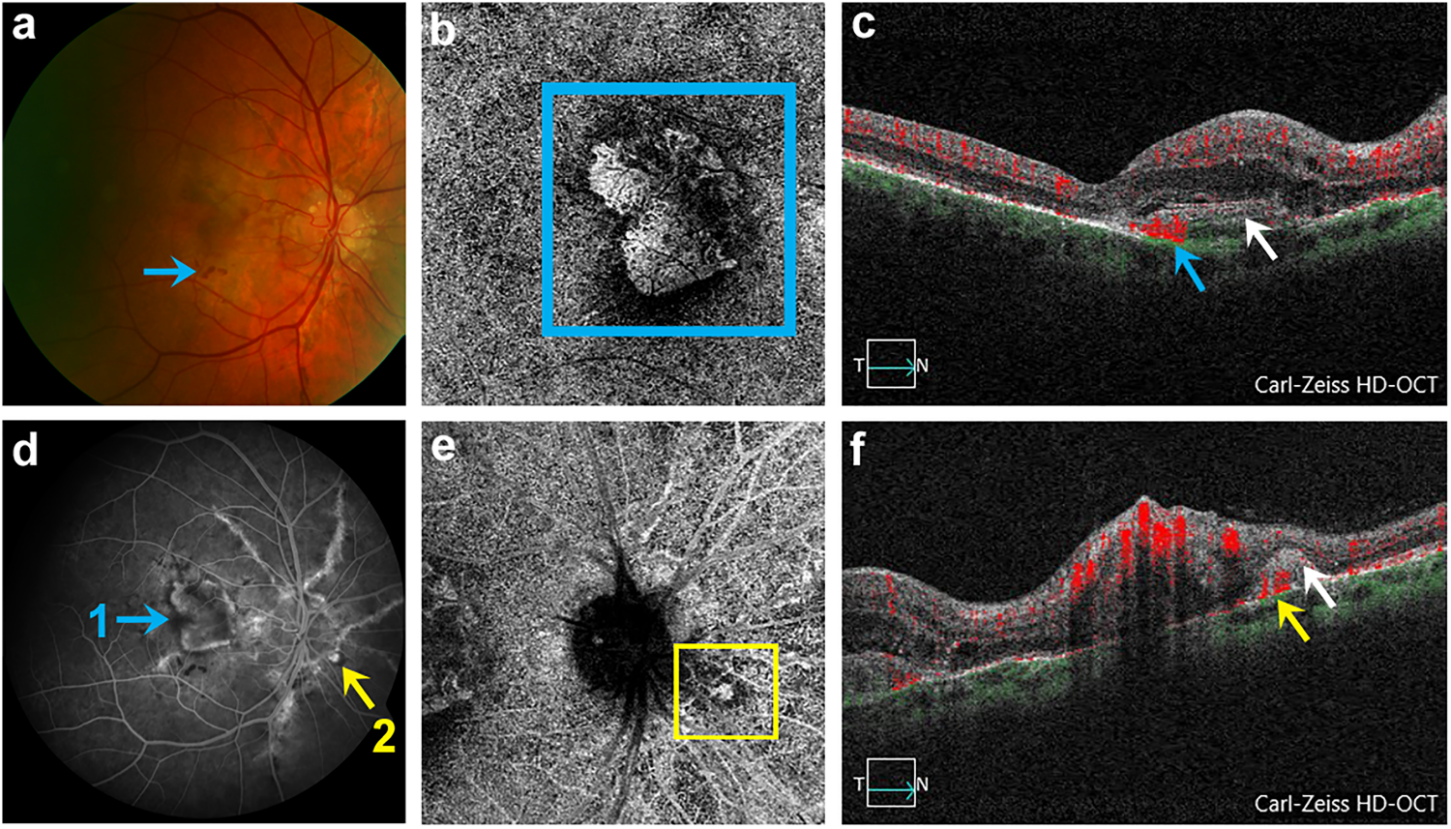
Right eye with angioid streaks (AS), 2 CNV (macular and juxtapapillary) and Optic nerve head drusen (ONHD) of study patient number 3 (a) Fundus with AS, ONHD, macular CNV and retinal hemorrhage (blue arrow). (b) OCTA of detected macular CNV in outer retina-chorio-capillaris (ORCC) segmentation (blue square). (c) Corresponding B-scan of macular CNV with blood flow registration (blue arrow). (d) FA revealing macular CNV (designated as 1, marked with blue arrow) and juxtapapillary CNV (designated as 2, marked with yellow arrow). (e) SD-OCTA representation of detected juxtapapillary CNV in ORCC segmentation (yellow square). (f) Corresponding B-scan of juxtapapillary CNV with blood flow registration (yellow arrow) and subretinal hyper-reflective material (SHRM) (white arrow).

Two included patients were diagnosed with PXE disease according to the Plomp criteria (Figures 1 and 2). However, four out of six patients declined genetic testing and therefore the disease may be underdiagnosed in this study cohort (Table 1). Associated findings were peau d'orange (2 patients), comet lesions (3 patients) and optic nerve head drusen (1 patient; Figure 3).

**Table 1.** Demographic details of the study cohort.

Study eyes / patients with secondary CNV, (n)	11 / 6
Gender, female / male (n)	2 / 4
Median age [years] (minimum—maximum)	48 (40–71)
Mean follow-up [weeks] (minimum - maximum)	76.4 (30–158)
Lens status, phakic (n)	9
Pseudoexanthoma elasticum (n)	2
Mean BCVA (logMAR) at baseline (SD)	0.49 ± 0.45
Mean BCVA (logMAR) at last visit (SD)	0.55 ± 0.51
**CNV detected by SD-OCTA**	
Type 1 / Type 2	4 / 9
**CNV detected by FA**	
CNV Type: n occult / classic	4 / 6

Abbreviations: FA: fluorescein angiography; SD: standard deviation

### Detection of CNV at baseline

Overall, 13 CNVs were detected in 11 study eyes, with bilateral CNV being observed in 5 patients. In addition, three patients presented with unilateral macular hemorrhage secondary to CNV. Eleven CNVs were located in the macular region (seven subfoveal and four extrafoveal) and two CNVs were located nasally adjacent to the optic disc (Table 2). According to the criteria described above, twelve CNVs were classified as active, all with SHRM and one as “quiescent” CNV (inactive treatment-naïve CNV) (Table 2).

**Table 2.** Clinical, fluorescein angiographic and optical coherence tomography angiographic characteristics of secondary CNV at baseline. A total of 13 CNV were detected in 11 study eyes and 12 of 13 CNV were graded as active according to SD-OCT, SD-OCT-A and FA findings.

Study Subject	CNV	Age	Eye	Treatment Status	CNV Location	CNV Type	SD-OCT and SD-OCTA Features	CNV Detectability by FA
**1**	1	53	R	naive	subfoveal	2, active	SHRM, IRF, ORCC-F	Yes
**1**	2	53	L	naive	subfoveal	2, active	SHRM, IRF, ORCC-F	Yes
**2**	3	71	R	pretreated	subfoveal	2, active	SHRM, IRF, SRF, ORCC-F	Yes
**2**	4	71	L	pretreated	subfoveal	2, active	SHRM, IRF, ORCC-F	Yes
**3**	5	48	R	naive	subfoveal	2, active	SHRM, IRF, SRF, ORCC-F	Yes
**3**	6	48	R	naive	juxtapapillary	2, active	SHRM, ORCC-F	No
**3**	7	48	L	naive	extrafoveal	2, active	SHRM, ORCC-F	No
**3**	8	48	L	naive	juxtapapillary	2, active	SHRM, ORCC-F	No
**4**	9	42	R	naive	extrafoveal	2, active	SHRM, IRF, SRF, ORCC-F	Yes
**5**	10	62	R	naive	extrafoveal	1, inactive	ORCC-F	Yes
**5**	11	62	L	naive	subfoveal	1, active	SHRM, SRF, ORCC-F	Yes
**6**	12	40	R	naive	subfoveal	1, active	SHRM, SRF, ORCC-F	Yes
**6**	13	40	L	naive	extrafoveal	1, active	SHRM, IRF, ORCC-F	Yes

Abbreviations: FA: fluorescein angiography; IRF: intraretinal fluid; L: left eye; ORCC-F: CNV finding in outer retina-chorio-capillaris segmentation with corresponding B-scans of OCTA; R: right eye; SHRM: subretinal hyper-reflective material; SRF: subretinal fluid.

Interestingly, ten CNVs (six classic and four occult) were detected by FA. A total of 13 CNVs were detected in 11 study eyes by SD-OCTA in the ORCC-segmentation without manual adjustment (9 eyes with type 2 CNV and 4 eyes with type 1 CNV) (Table 2). All eyes with an active CNV showed SHRM on SD-OCT and were treated with intravitreal anti-VEGF injections according to a PRN protocol.

### Treatment and follow-up

On average, each study eye received 9.4 anti-VEGF injections (either bevacizumab or ranibizumab) during a mean follow-up period of 76 weeks (Table 1). Best-corrected visual acuity (BCVA) improved in 5 eyes, remained unchanged in 4 eyes, and worsened in two eyes. Unfortunately, vision deterioration in one eye was +1.6 logMAR due to the development of fibrosis after CNV refractory to therapy. Mean BCVA at the last visit was 0.55 ± 0.51 logMAR. During the follow-up period a quiescent CNV converted to an active form, therefore a re-treatment with anti-VEGF regimen was initiated. In the rest of the eyes CNVs regressed and no recurrence was observed.

## Discussion

In this study we found that multimodal imaging with SD-OCT, SD-OCTA and FAF is a reliable diagnostic tool for the detection of secondary CNV in AS. Due to defects in the BM-RPE complex, the identification of secondary CNV with SD-OCTA showed favorable results compared to FA, especially in eyes with AS. In our study, SD-OCTA detected a total of 13 CNVs in 11 eyes compared to 10 CNVs by FA. Of the 13 CNV, 12 were active and needed to be treated. Prompt treatment and monitoring of CNV preserved visual acuity over a mean follow-up of 76 weeks in our cohort. For therapy monitoring, SD-OCTA and SD-OCT were shown to be a valuable diagnostic modality.

Detection of secondary CNV in AS is crucial for early treatment initiation. Only timely treatment with anti-VEGF agents can help preventing vision loss in these patients. In addition, disease monitoring is essential for CNV secondary to AS, as they are often refractory to therapy and show a high recurrence rate.^3,10,11,14^ In this context, multimodal imaging including both SD-OCTA and SD-OCT is gaining ground in the everyday clinical practice, as one can adequately identify CNV secondary to AS in a non-invasive and time-saving manner compared to FA. The choice of multimodal imaging is multifaceted, and the determination of an appropriate imaging modality should be suitable both for confirming the diagnosis of the underlying disease as well as for treatment initiation. With the advent of SD-OCTA, a risk-free imaging alternative is now available for detecting and monitoring secondary CNV. This is particularly beneficial for patients who have experienced previous adverse events or for whom FA is contraindicated.

With a total of 13 CNVs detected by SD-OCTA in 11 study eyes compared to 10 CNVs detected by FA, our study showed that SD-OCTA was more reliable than FA in detecting CNVs. Furthermore, the combination of SD-OCT and SD-OCTA provided more information to assess CNV activity than conventional FA, due to OCT biomarkers and supplemental B-scans of SD-OCTA. Similar to our data, SD-OCTA has been previously applied for the detection of CNV secondary to AS. With SD-OCTA, the grading of CNV into active and non-active has been demonstrated.^21^ De Zaeytijd and colleagues detected the presence of CNV secondary to AS in 87.5% of eyes using SD-OCTA. In the remaining eyes, detection was not possible due to poor image quality of the SD-OCTA scans compared to FA.^29^ Therefore, we excluded patients with media opacities from this study.

Although multimodal imaging offers many advantages, FA seems to remain an indispensable component in the diagnosis of secondary CNV in AS and represents a diagnostic gold standard.^30^ Despite the widespread use of fluorescein angiography (FA) and its ability to detect dynamic patterns of dye transit and leakage with ultra-widefield images, some authors recommend that indocyanine green angiography is even superior, especially for the detection of type 1 CNV.^30^ However, there are many studies suggesting a high sensitivity and specificity for the identification of type 1 and type 2 CNV on SD-OCTA, especially the non-exudative type 1 CNV.^30,31^ In our study, the majority of CNVs detected were of type 2 (69.2%), which results from defects in the BM-RPE complex.^10^ On FA, these defects appear as hyperfluorescent areas and may compromise the detection of secondary CNV in AS.^20,32^

Several studies have shown the advantages of SD-OCT and SD-OCTA, including reliable visualization of neovascularization in greater detail than with FA.^33–36^

In this study and in agreement with the literature, especially CNV secondary to AS is easily accessible to the examiner with SD-OCTA as it is usually localized in the posterior pole (macular or peripapillary) and consists of a limited area.^1^ In addition to CNV detection, SD-OCTA can illustrate various CNV growth patterns through three-dimensional imaging of vascular structure and assessing CNV activity. This can aid in monitoring therapy during intravitreal treatment.^35^ It should be noted that SD-OCTA is a relatively new and not fully standardized imaging technique compared to FA. In particular, imaging artefacts and segmentation errors are common in daily use and must be taken into account. In addition, SD-OCTA patterns are still under discussion and no consensus has been reached, especially regarding treatment decisions. However, a clinically experienced retinal specialist can reliably detect CNV with SD-OCTA when using the standardized segmentation line, especially the ORCC segmentation for secondary CNV.

This study has some limitations including the small number of cases, which limits the power of the results. As this is a rare condition, the small number of cases is due to the low prevalence of CNV secondary to AS during this period. In addition, the retrospective nature and lack of a control group limit the data. The heterogeneity of the baseline in our group and the use of both eyes of the same patient were also taken into account. It is also important to stress the potential differential diagnosis of rare retinal diseases based on outer retinal inflammation, such as PXE-associated acute retinopathy or punctate inner choroidopathy-like reactions that may occur as a result of PXE disease with a non-neovascular fundus.^37–39^

In conclusion, SD-OCTA in combination with SD-OCT seems to be an excellent tool for early detection and monitoring of AS and its neovascular complications. Further studies in these rare retinal entities are warranted.
